# Comprehensive analysis of immune cell landscapes revealed that immune cell ratio eosinophil/B.cell.memory is predictive of survival in sepsis

**DOI:** 10.1186/s40001-023-01506-8

**Published:** 2023-12-05

**Authors:** Lei Wang, Guoan Zhang, Wenjie Sun, Yan Zhang, Yi Tian, Xiaohui Yang, Yingfu Liu

**Affiliations:** 1 Microbiology and Immunology Department, Cangzhou Medical College, Cangzhou, 061001 Hebei China; 2 Science and Technology Experiment Center, Cangzhou Medical College, Cangzhou, 061001 Hebei China; 3 University Nanobody Application Technology Research and Development Center of Hebei Province, Cangzhou, 061001 Hebei China; 4 Cangzhou Nanobody Technology Innovation Center, Cangzhou, 061001 Hebei China

**Keywords:** Septic shock, Acute respiratory distress syndrome (ARDS), Influenza, Receiver operating characteristic (ROC), Intensive care unit (ICU)

## Abstract

**Background:**

Immune dysregulation is a feature of sepsis. However, a comprehensive analysis of the immune landscapes in septic patients has not been conducted.

**Objectives:**

This study aims to explore the abundance ratios of immune cells in sepsis and investigate their clinical value.

**Methods:**

Sepsis transcriptome data sets were downloaded from the NCBI GEO database. The immunedeconv R package was employed to analyze the abundance of immune cells in sepsis patients and calculate the ratios of different immune cell types. Differential analysis of immune cell ratios was performed using the *t* test. The Spearman rank correlation coefficient was utilized to find the relationships between immune cell abundance and pathways. The prognostic significance of immune cell ratios for patient survival probability was assessed using the log-rank test. In addition, differential gene expression was performed using the limma package, and gene co-expression analysis was executed using the WGCNA package.

**Results:**

We found significant changes in immune cell ratios between sepsis patients and healthy controls. Some of these ratios were associated with 28-day survival. Certain pathways showed significant correlations with immune cell ratios. Notably, six immune cell ratios demonstrated discriminative ability for patients with systemic inflammatory response syndrome (SIRS), bacterial sepsis, and viral sepsis, with an Area Under the Curve (AUC) larger than 0.84. Patients with a high eosinophil/B.cell.memory ratio exhibited poor survival outcomes. A total of 774 differential genes were identified in sepsis patients with a high eosinophil/B.cell.memory ratio compared to those with a low ratio. These genes were organized into seven co-expression modules associated with relevant pathways, including interferon signaling, T-cell receptor signaling, and specific granule pathways.

**Conclusions:**

Immune cell ratios eosinophil/B.cell.memory and NK.cell.activated/NK.cell.resting in sepsis patients can be utilized for disease subtyping, prognosis, and diagnosis. The proposed cell ratios may have higher prognostic values than the neutrophil-to-lymphocyte ratio (NLR).

**Supplementary Information:**

The online version contains supplementary material available at 10.1186/s40001-023-01506-8.

Sepsis poses a significant global clinical challenge and places a considerable economic burden on intensive care units (ICUs). It is a leading cause of multi-organ failure and mortality in ICUs [1]. Initially, systemic inflammatory response syndrome (SIRS) was defined as a non-infectious clinical syndrome characterized by a widespread inflammatory response throughout the body, such as pancreatitis or surgical trauma [2]. However, over the years, sepsis definitions and clinical criteria have evolved, leading to the introduction of the Sepsis-3 criteria in 2016. The Sepsis-3 criteria provide a more accurate and clinically relevant framework for diagnosing and managing sepsis. According to the current Sepsis-3 definition, sepsis is considered a life-threatening immune response to infection that leads to organ dysfunction [3]. The etiology of sepsis is diverse, ranging from bacterial infections to viral causes, such as influenza, pneumonia, COVID-19, and even food poisoning [4–7].

Sepsis is a life-threatening physiological condition triggered by an immune response dysregulation in the presence of infection and is often associated with significant mortality [8, 9]. The molecular mechanisms underlying sepsis are still being investigated [9]. Sepsis is a complex disease influenced by factors, such as infection types and genetic backgrounds [10]. The complexity of sepsis arises from the diverse array of pathogens that can trigger the condition, including bacterial, viral, and fungal infections, each with distinct pathogenic mechanisms and host responses [11]. In addition, the genetic makeup of individuals plays a significant role in determining their susceptibility to sepsis and the subsequent immune response [12]. Traditional biomarkers such as C-reactive protein (CRP), procalcitonin (PCT), interleukin 8 (IL8), and NLR have shown limited diagnostic performance [13, 14]. Biomarkers such as CRP, PCT, and IL8 lack specificity for sepsis. They can also be elevated in non-infectious inflammatory conditions, making it challenging to differentiate sepsis from other inflammatory states [9]. The levels of CRP, PCT, and IL8 can take time to rise significantly following the onset of infection or sepsis. This delay in their elevation can result in delayed diagnosis. However, cellular-level biomarkers are still rare. It is necessary to develop novel biomarkers for disease diagnosis and prognosis.

Immune cell ratios, which reflect the intricate interplay of various immune cell populations, have emerged as potential candidates for unraveling sepsis heterogeneity and predicting clinical outcomes [15, 16]. However, few studies have explored the role of immune cell ratios in sepsis, and their clinical implications remain largely unexplored. With the advent of high-throughput transcriptome data and sophisticated computational tools, deconvolving cell abundances from transcriptomic data has become increasingly feasible [17, 18]. Such approaches can provide valuable insights into the immune cell landscape and its alterations in sepsis at a large scale [19].

The traditional approach to characterizing immune cell populations in tissues involves techniques, such as flow cytometry or immunohistochemistry, which require the isolation and labeling of specific cell types for analysis. While these methods provide valuable experimental data, they are often limited by factors, such as sample availability, cost, and technical constraints [20]. Cell-type Identification By Estimating Relative Subsets of RNA Transcripts (CIBERSORT) is a computational method that addresses these limitations by leveraging the power of transcriptomic data. CIBERSORT can estimate the abundance of different immune cell populations within a heterogeneous tissue sample using gene expression profiles [20].

Here, we first proposed the utilization of immune cell ratios as novel biomarkers for sepsis diagnosis, subtyping, prognosis, and treatment. We applied the method to sepsis and identified specific ratios associated with sepsis, survival outcomes, and infection types (Fig. 1). Our analysis may provide important information for clinical applications.

**Fig. 1:**
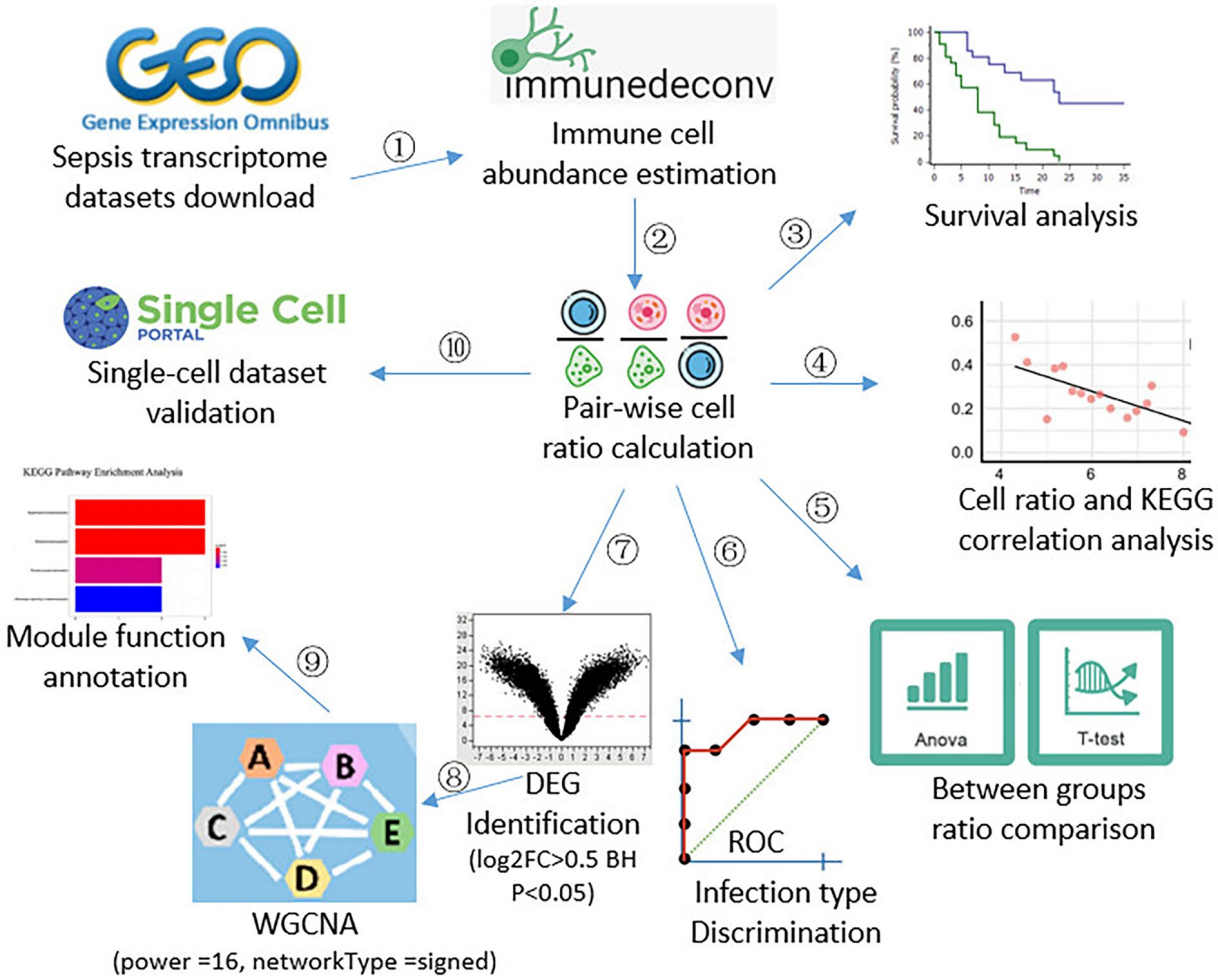
Schematic diagram shows the workflow of the study.① Three blood data sets were used for deconvolving immune cell abundances. ② Immune cell ratios were calculated for each data sets. ③, ④, ⑤, and ⑥ Immune cell ratios were used for differential analysis, survival analysis, KEGG pathway correlation analysis, and discrimination performance analysis. ⑦ Differential expressing genes (DEGs) identification between samples with high and low eosinophil/B.cell.memory ratios. ⑧ Weighted gene co-expression network analysis (WGCNA) based on DEGs. ⑨ Function annotation of gene co-expression module. ⑩ Key cell ratios were validated by Single Cell Portal database. ROC: receiver operating characteristic

## Materials and methods

### Data sets download and sample selection

Sepsis-related data sets were obtained from the National Center for Biotechnology Information Gene Expression Omnibus (NCBI GEO). Specifically, GSE185263, which consists of 392 whole blood samples (44 healthy control samples and 348 sepsis samples) and utilizes Illumina HiSeq 2500 technology, was utilized for the analysis of sepsis immune cell proportion changes [21]. As GSE185263 is based on state-of-the-art RNA-Seq transcriptome technology, it was suitable for this purpose. For the identification of survival-related immune cell ratios, GSE65682 was employed. This data set includes 802 sepsis blood samples from critically ill patients and contains patient survival information. GSE65682 utilizes the Affymetrix Human Genome U219 platform [6]. To test the association between immune cell ratios and different infection types, GSE63990 was used. This data set comprises 273 samples, including 88 samples of systemic inflammatory response syndrome (SIRS), 115 samples of viral acute respiratory infections, and 70 samples of bacterial acute respiratory infections. GSE63990 utilizes the Affymetrix Human Genome U133A 2.0 platform [22]. In addition, for the analysis of bacterial sepsis at the single-cell level, relevant data were retrieved from the website https://singlecell.broadinstitute.org/ [23].

### Transcriptome data analysis

The expression matrix was analyzed by CIBERSORT, quanTIseq, and EPIC algorithms integrated in the R immunedeconv package for immune cell abundance analysis [20]. A relative immune cell abundance matrix was obtained. Differential cell ratios were identified by the t.test function. For the analysis of differential genes, we utilized the limma package. The criteria for the differential gene were log2(FoldChange) > 0.5 and Benjamini–Hochberg *P* < 0.05. To identify co-expression modules, we performed the unsupervised WGCNA analysis by following the guidelines outlined in the package manual [24]. Key parameters were set as follows: power = 16, networkType = signed, minModuleSize = 20, and deepSplit = 4. As we focused on DEGs, WGCNA was used as a dimension–reduction method to partition 6492 most variable genes into modules, and 774 DEGs were mapped to modules for functional annotation. In a gene co-expression network, a module refers to a group of genes that exhibit similar expression patterns across different samples. These co-expressed genes are believed to be functionally related or involved in similar biological processes. The gene module functional enrichment in terms of GO and pathways was conducted using the clusterProfiler package [25]. The differential expression between modules was analyzed by the Student’s *t* test using the module eigengene (ME) values. For quantifying the expression of KEGG pathways, we employed the fgsea package with the following parameters: minSize = 15, maxSize = 500, and scoreType = "pos".

### Statistical analysis

Student’s *t* test was used to compare the mean difference of cell ratios between sepsis and control groups in GSE185263, as the sample size is large. Analysis of variance (ANOVA) was used to compare the mean of cell ratio in multiple groups, including SIRS, bacterial, and viral sepsis. The correlation between the two variables was calculated by Pearson or Spearman correlation coefficient for the relationship between KEGG pathways and immune cell ratios. Patients were split into two groups by the median value. Survival difference between the two groups was tested by log-rank test. All the significant thresholds were set as *P* < 0.01 unless otherwise specified. Survival analysis based on immune cell proportions was performed by R survival package with *P* < 0.01. ROC curves were plotted by the R pROC package. The schematic diagram of our analysis workflow is provided in Fig. 1.

## Results

### Analysis of sepsis immune cell proportion changes

**Fig. 2:**
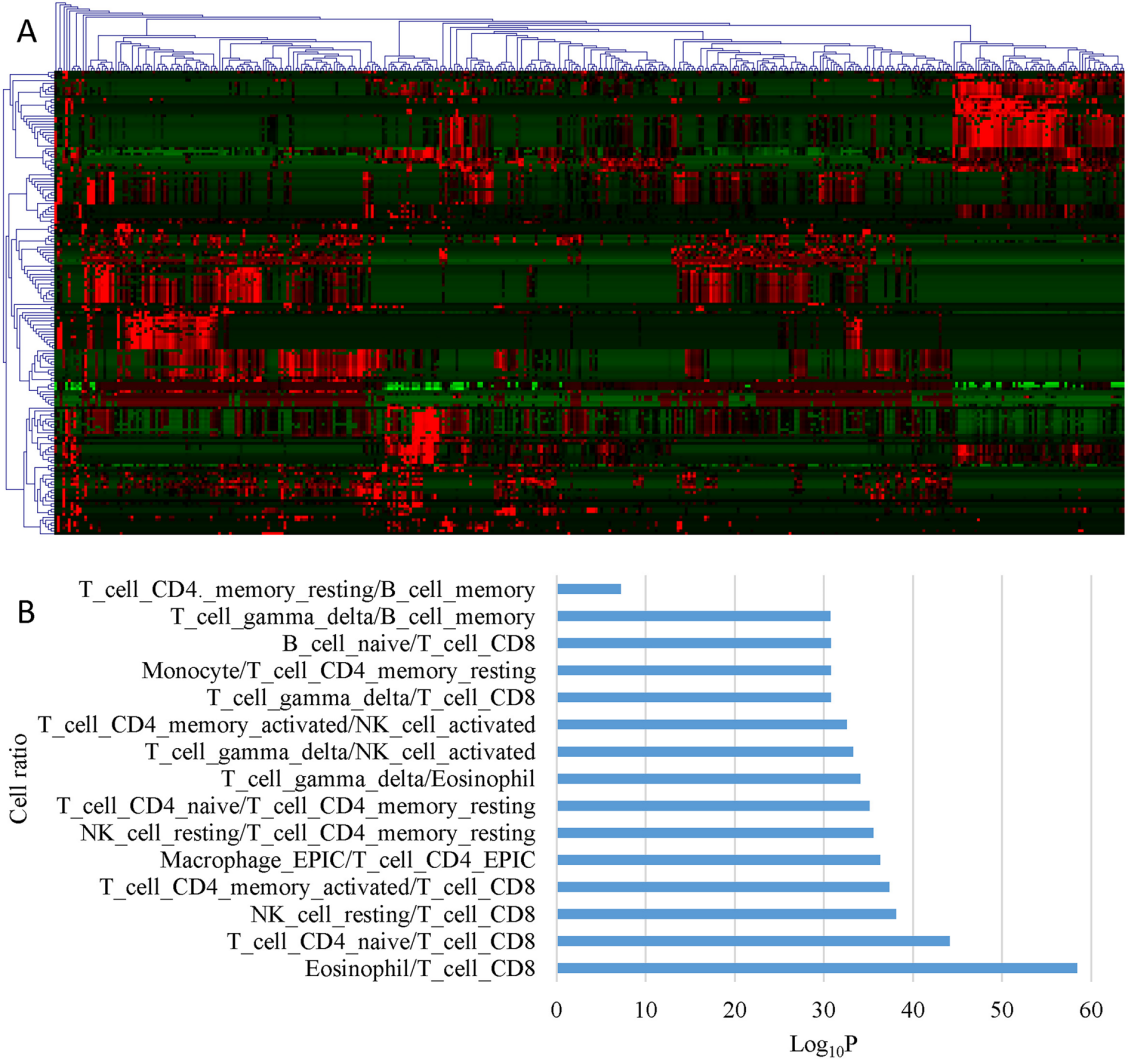
Immune cell ratios are altered in sepsis data set GSE185263. **A** Clustering heat map of immune cell ratios in healthy control and sepsis patients. **B** Bar plot of the top 15 significantly up-regulated cell ratios

Compared to healthy control, 171 pairs of immune cell ratios were found to be altered at a significance level of *P* < 5E−5 in the sepsis data set GSE185263 (Fig. 2A). Among these, 15 immune cell ratios with the most significant *P* value were mainly associated with T cells (Fig. 2B). This suggests a dramatic alteration in the acquired immune system during sepsis. Results also showed immune cell ratios had different expression patterns between sepsis and normal subjects (Fig. 2A). Moreover, among sepsis patients, the expression patterns of immune cell ratios also differed, possibly due to inherent disease heterogeneity, indicating the presence of different subtypes.

### Relationship between immune cell proportion and 28-day survival

**Fig. 3:**
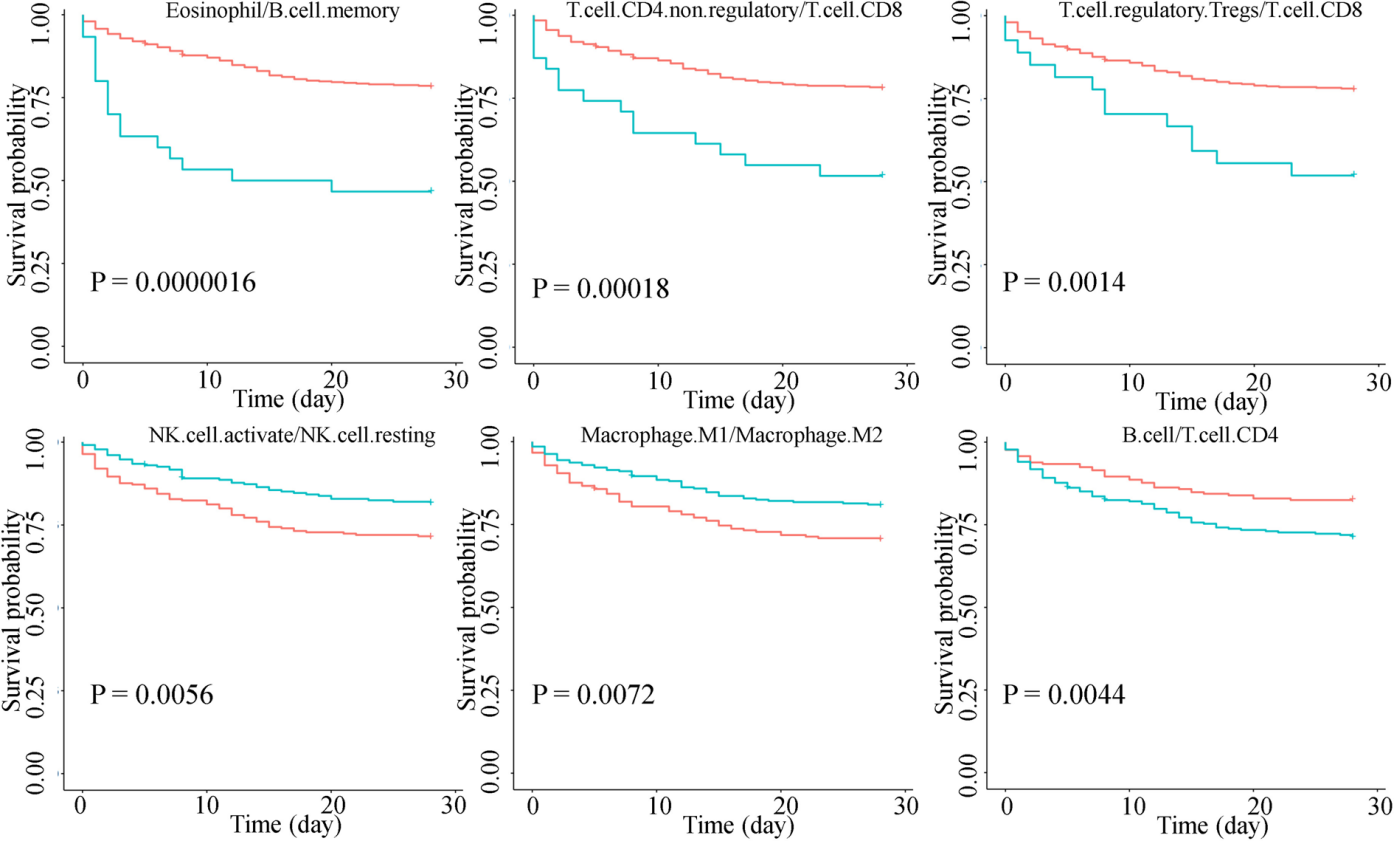
Survival analysis by immune cell ratio based on data set GSE65682. Red curves denote a low immune cell ratio and green curves denote a high immune cell ratio. The *P* value was calculated by log-rank test

Survival analysis of critically ill patient data set GSE65682 showed a correlation between immune cell proportions and survival (Fig. 3). 40 immune cell ratios were found to be correlated with 28-day survival (*P* < 0.01). Notably, immune cell ratios eosinophil/B.cell.memory, T.cell.CD4.non.regulatory/T.cell.CD8, Tregs/T.cell.CD8, NK.cell.activated/NK.cell.resting, Macrophage.M1/Macrophage.M2, and B.cell/T.cell.CD4 showed significant associations with survival. Therefore, activated NK proportion, CD8 T cell, and M1 macrophage proportions were positively associated with survival rates. After adjusting for gender and age, all six cell ratios were still significant for survival prognosis. Among the six cell ratios, ratios eosinophil/B.cell.memory and NK.cell.activated/NK.cell.resting were still significant in survival prognosis after adjusting for gender, age, pneumonia, thrombocytopenia, and diabetes mellitus.

### Relationship between KEGG pathways and immune cell ratios

The correlation analysis between immune cell ratios and the expression of the KEGG pathways showed significant associations between specific immune cell ratios and KEGG pathways. For example, the T-cell CD4 memory resting/T-cell follicular helper ratio displayed the highest positive correlation with the T-cell receptor signaling pathway (*R* = 0.68, *P* < 0.01), which was down-regulated in sepsis patients. On the other hand, the eosinophil/T-cell CD8 ratio showed the highest negative correlation with primary immunodeficiency (*R* = − 0.70, *P* < 0.01), which was down-regulated in sepsis. Besides, each pathway exhibited the highest correlation value with specific cell ratios. For example, the Toll-like receptor signaling pathway had the highest correlation with the neutrophil/macrophage M2 ratio (*R* = 0.66, *P* < 0.01), and it was up-regulated in sepsis. Complement and coagulation cascades had the highest correlation with the neutrophil/Tregs ratio (*R* = 0.61, *P* < 0.01), and it was up-regulated in sepsis. Chronic myeloid leukemia had the highest correlation value with the T-cell CD4 memory resting/macrophage M1 ratio (*R* = 0.60, *P* < 0.01), and it was down-regulated in sepsis. Colorectal cancer had the highest correlation with the T-cell CD4 memory resting/mast-cell resting ratio (*R* = 0.60, *P* < 0.01), and it was down-regulated in sepsis. Melanogenesis had the highest correlation with the T-cell CD4 memory resting/macrophage M2 ratio (*R* = 0.60, *P* < 0.01), and it was down-regulated in sepsis. The NOD-like receptor signaling pathway had the highest correlation with the T-cell CD8/mast-cell resting ratio (*R* = − 0.68, *P* < 0.01), and it was up-regulated in sepsis. Progesterone mediated oocyte maturation had the highest correlation with the T-cell CD4 memory resting/eosinophil ratio (*R* = 0.65, *P* < 0.01), and it was down-regulated in sepsis. Small cell lung cancer had the highest correlation with the T-cell CD4 memory resting/macrophage M1 ratio (*R* = 0.65, *P* < 0.01), and it was down-regulated in sepsis. The VEGF signaling pathway had the highest correlation with the T-cell CD4 memory resting/eosinophil ratio (*R* = 0.61, *P* < 0.01), and it was down-regulated in sepsis. Finally, the Wnt signaling pathway had the highest correlation with the macrophage/T-cell CD4 ratio (*R* = − 0.61, *P* < 0.01), and it was down-regulated in sepsis.

### Relationship between the immune cell ratios and the type of infection

**Fig. 4:**
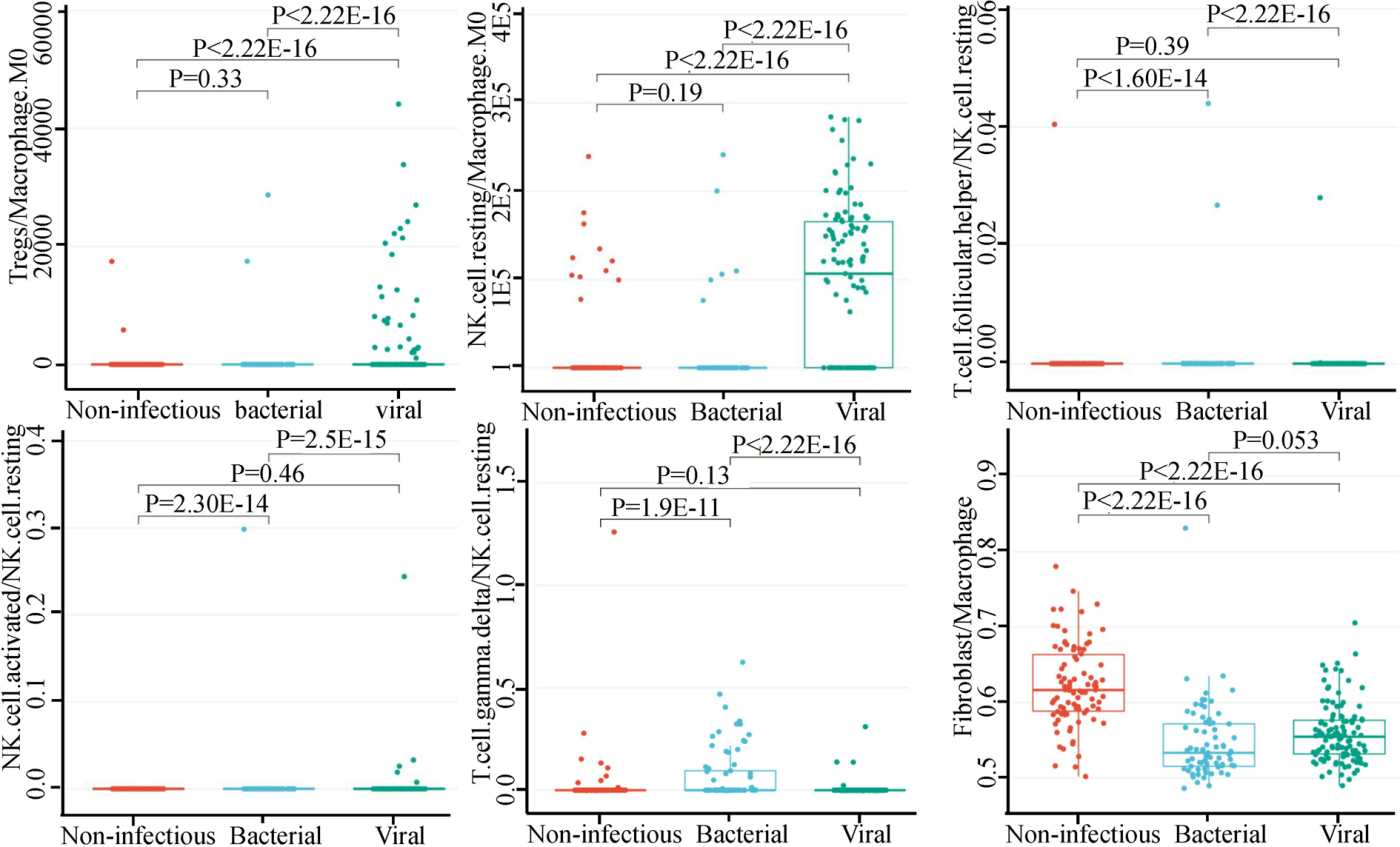
Immune cell ratios were differentially expressed among SIRS, bacterial, and viral sepsis in data set GSE63990. Six cell ratios including Tregs/macrophage M0, NK-cell resting/macrophage M0, T.cell.follicular.helper/NK.cell.resting, NK.cell.activated/NK.cell.resting, T.cell.gamma.delta/NK.cell.resting, and fibroblast/macrophage were differentially expressed among SIRS, bacterial, and viral sepsis as tested by ANOVA

**Fig. 5:**
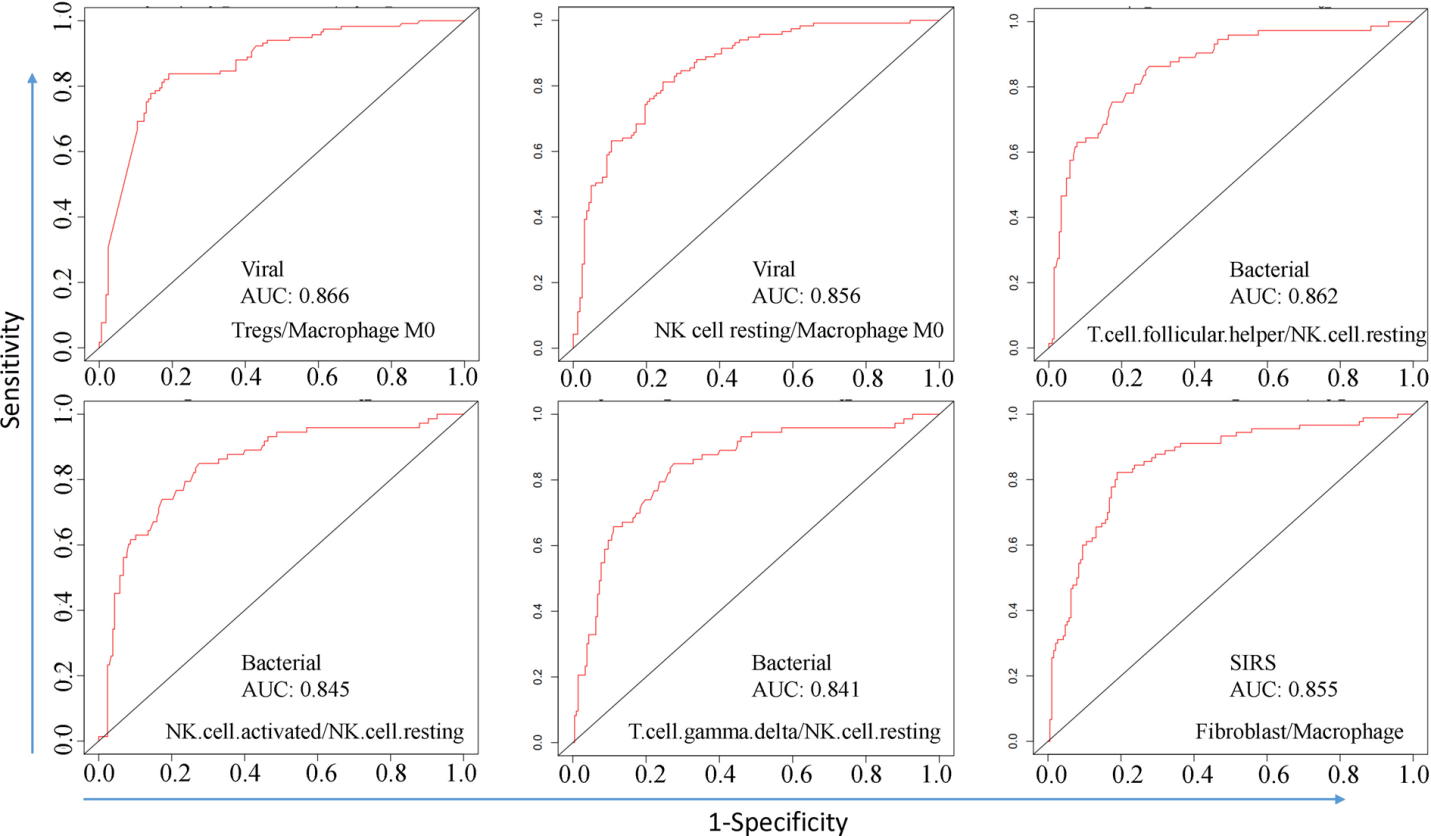
Differentiating SIRS, bacterial, and viral sepsis using immune cell ratio based on data set GSE63990. Six ROC curves show the performance of six cell ratios, including Tregs/macrophage M0, NK-cell resting/macrophage M0, T.cell.follicular.helper/NK.cell.resting, NK.cell.activated/NK.cell.resting, T.cell.gamma.delta/NK.cell.resting, and fibroblast/macrophage in differentiating viral sepsis, bacterial sepsis, and SIRS

Using immune cell ratios to distinguish different types of infections in the data set GSE63990, we observed that the type of infection was significantly related to specific immune cell ratios. For example, the Tregs/macrophage M0, NK-cell resting/macrophage M0 ratios showed differential expression among non-infectious, bacterial, and viral sepsis (Fig. 4). Notably, high expression of these two ratios effectively differentiated viral infection from other types of infections, as indicated by the area under the curve (AUC) values of 0.866 and 0.856, respectively (Fig. 5). Besides, the high fibroblast/macrophage ratio could differentiate SIRS from other infections with an AUC of 0.855. High T-cell follicular helper/NK-cell resting, NK-cell-activated/NK-cell resting, and T-cell gamma delta/NK-cell resting ratios could differentiate bacterial infection from other infections with AUCs of 0.862, 0.845, and 0.841, respectively. Analyzing the difference in immune cell ratios between viral and bacterial sepsis, we found that the two most significant cell ratios were Macrophage M0/NK-cell resting (up-regulated in bacterial sepsis, *P* = 4E−13) and monocyte/NK-cell resting (up-regulated in bacterial sepsis, *P* = 4E−13).

### Differential gene expression analysis reveals key pathways in sepsis with high eosinophil/B.cell.memory ratio

**Fig. 6:**
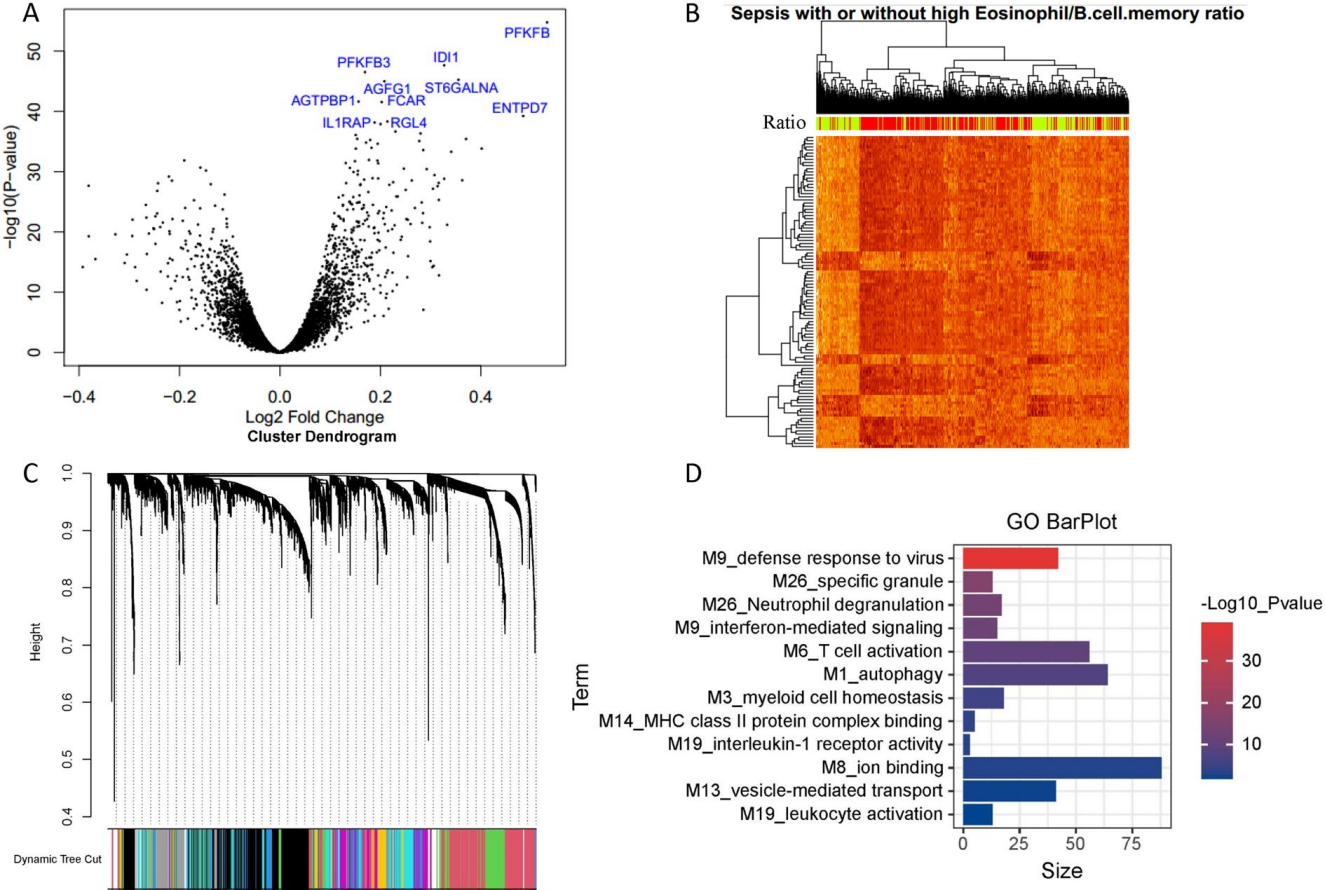
Differential gene expression analysis in two groups of sepsis samples with high eosinophil/B.cell.memory ratio vs. low eosinophil/B.cell.memory ratio based on data set GSE65682. **A** Volcano plot shows the differential gene expression between the two groups. The top ten most significant genes were labeled with their gene names. **B** Clustering heat map of the 774 differential genes reveals the distinct gene expression pattern between the two groups. The rows represent the differential genes and the columns represent the sepsis samples. **C** Cluster dendrogram shows the partition of 6492 genes into 21 gene modules with different colors. The color bar indicates the assignment of genes to a module. **D** Bar plot showing the functional enrichment result of the 9 identified modules. These modules with mapped DEGs are M1, M3, M6, M8, M9, M13, M14, M19, and M26

To check if there is any biological relevance between the two groups of sepsis patients, we performed differential gene expression analysis. A total of 774 differential genes were identified in samples with high expression of eosinophil/B.cell.memory ratio compared to those with low ratios (Fig. 6A). The clustering analysis demonstrated distinct gene expression patterns between the two groups (Fig. 6B). Furthermore, genome-wide gene co-expression analysis of all samples in GSE65682 partitioned 6492 genes into 21 modules (Fig. 6C). Functional enrichment analysis revealed that these modules were associated with specific biological pathways (Fig. 6D). DEGs were mainly distributed 9 modules. Modules M1, M13, M19, and M26 were significantly up-regulated, while M3, M6, M8, M9, and M14 were significantly down-regulated in sepsis with high eosinophil/B.cell.memory ratio. The most significant up-regulated module was M1 (annotated as autophagy), while the most down-regulated module was M9 (annotated as interferon-mediated signaling). All DEGs and genes in all modules are provided in Additional file 1: Tables S1, S2.

### Validation of key cell ratios in single-cell sepsis data set by gene ratios

**Fig. 7:**
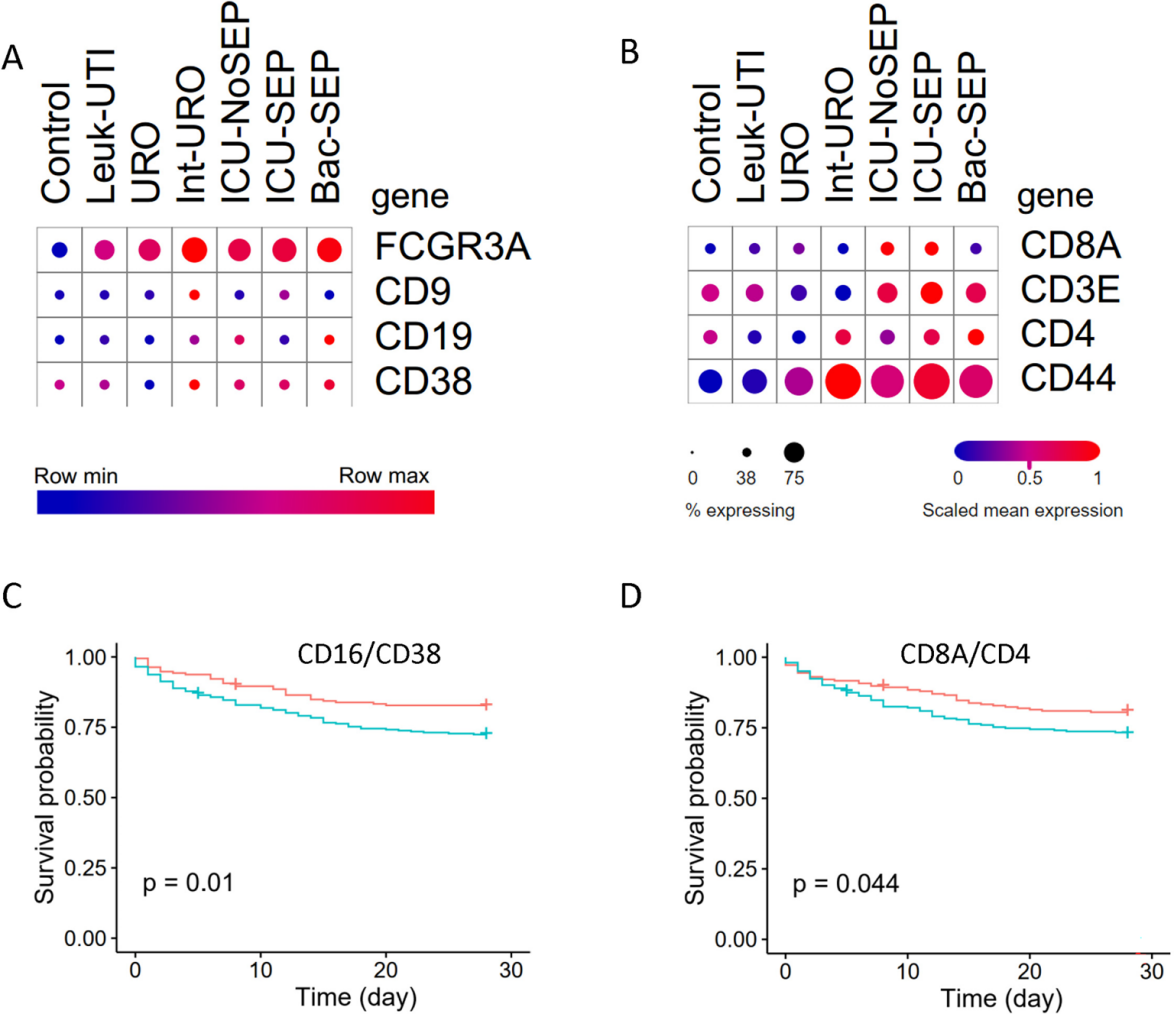
Two cell ratios were validated in the single-cell data set. **A** Expression of marker genes for eosinophil and memory B cell in control and bacterial sepsis. The dot size represents the percent of cells that express the gene. **B** Expression of marker genes for non-regulatory CD4 T cell and CD8 T cell in control and bacterial sepsis. **C** Survival analysis based on marker gene ratio of eosinophil and memory B cell in data set GSE65682. **D** Survival analysis based on marker gene ratio of non-regulatory CD4 T cell and CD8 T cell in data set GSE65682. The green line and red line denote a high and low value of expression ratio, respectively

To validate the association of these cell ratios with sepsis, we used the single-cell data set from the Broad Institute. We focused on the top 2 most significant cell ratios, namely, eosinophil/B.cell.memory and T.cell.CD4.non.regulatory/T.cell.CD8. We found that eosinophil/B.cell.memory ratio was up-regulated in sepsis, whereas T.cell.CD4.non.regulatory/T.cell.CD8 was down-regulated in sepsis (Fig. 7A, B). We further used the marker gene ratios from data set GSE65682 for survival analysis. When stimulated in vitro with platelet-activating factor or IFNgamma, blood eosinophils exhibit surface expression of CD16 [26]. We found that higher gene ratios of CD16/CD38 and CD8A/CD4 were associated with poorer survival outcomes (Fig. 7C, D). These results indicate the possible protective roles of memory B cells and CD8 T cells in sepsis.

We observed that the gene ratio CD16/CD38 had a weaker association with survival than the cell ratio. The reason for the discrepancy may be that the gene ratio CD16/CD38 is not as specific for sepsis as the cell ratio. We found that the gene ratio CD16/CD38 was down-regulated in sepsis and was not associated with the sequential organ failure assessment score (SOFA), which is an indicator of sepsis severity (Additional file 2: Fig. S1A, B). Lower gene ratio CD16/CD38 can be used for the viral sepsis diagnosis (Additional file 2: Fig. S1C). It is also possible that the gene ratio CD16/CD38 is more sensitive to other factors, such as the type of infection (Additional file 2: Fig. S1D).

### Comparison of the prognostic values of cell ratios and neutrophil-to-lymphocyte ratio (NLR)

**Fig. 8:**
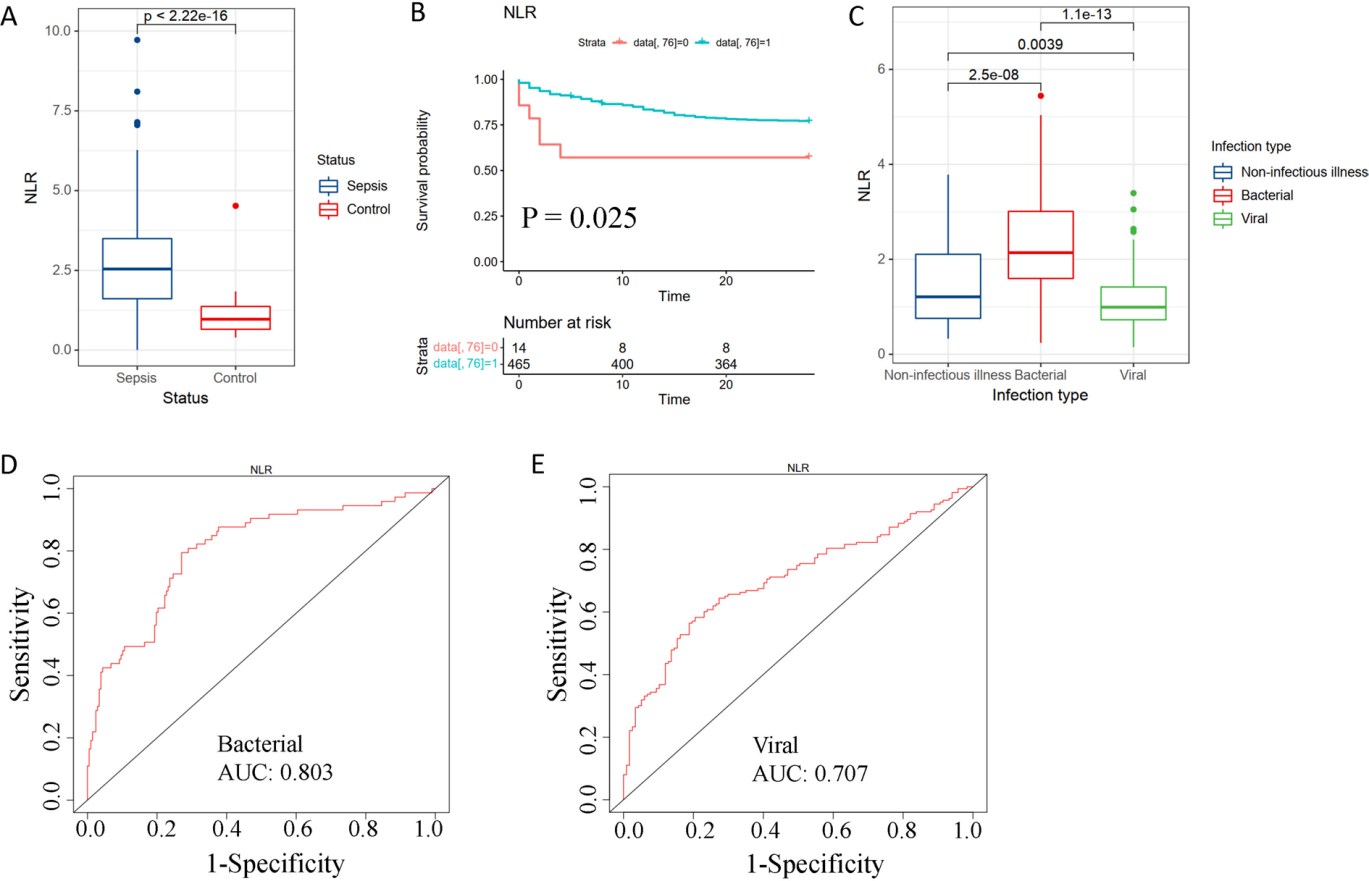
Prognostic value of neutrophil-to-lymphocyte ratio (NLR) in sepsis. **A** Boxplot for NLR in control and sepsis subjects of data set GSE185263. **B** Survival analysis of NLR in data set GSE65682. Green line: NLR > 1. Red line: NLR < 1. **C** Analysis of variance (ANOVA) of NLR in different infection types, including non-infectious illness, bacterial infection, and viral infection in data set GSE63990. **D**, **E** ROC analysis of NLR in bacterial and viral sepsis diagnosis in data set GSE63990. AUC: area under the curve. The NLR was calculated using the CIBERSORT results

NLR is a prognostic marker in sepsis. To compare the prognostic values of cell ratios and NLR, we summed up the lymphocyte cell proportions and calculated the NLR using the CIBERSORT results. NLR was up-regulated in sepsis (Fig. 8A). Survival analysis revealed that NLR was marginal in prognosis (Fig. 8B), indicating the superior performance of cell ratios than NLR. Comparison among groups by ANOVA showed that NLR was up-regulated in bacterial sepsis but down-regulated in viral NLR (Fig. 8C), indicating the different mechanisms of bacterial and viral sepsis. ROC analysis of NLR suggested that its diagnosis ability is no better than cell ratios (Fig. 8D, E). These results indicate that cell ratios have higher prognostic value.

## Discussion

Currently, only a few studies have utilized immune cell ratios for prognostic purposes in sepsis [27, 28]. This research presents a comprehensive analysis of immune cell ratio changes in sepsis using cell-level transcriptome data analysis. We explored the relationship between immune cell ratios and survival, the association between immune cell ratios and KEGG pathways, and the correlation between immune cell ratios and the type of infection. This study represents the first systematic analysis of immune cell ratios in sepsis, offering valuable insights for understanding the mechanisms, diagnosis, subtyping, and treatment of sepsis.

Existing research primarily focuses on analyzing gene sets, such as differential genes or co-expressed genes [29]. In contrast, our study systematically examined the expression changes of immune cell ratios in sepsis. Notably, the identified differential immune cell ratios are in line with existing literature. All the identified cell ratios are related to T cells, consistent with the phenomenon of sepsis-induced T-cell immune paralysis, characterized by reduced T-cell activity and numbers [11]. Eosinophil has been considered useful for the prognosis of severe sepsis, and its number has decreased [30]. In our study, we found that the eosinophil/T-cell CD8 ratio is up-regulated in sepsis, suggesting that the reduction of CD8 T cells is greater than that of eosinophils. It has been reported that the T-cell CD4 naïve number decreases and its function weakens, and its ability to respond to pathogens decreases in sepsis [31]. T-cell CD4 naïve/T-cell CD4 memory resting can affect CD4 T-cell phenotype [31]. B-cell memory count in sepsis is significantly down-regulated, which can cause the suppression of immune response [32]. We found T-cell gamma delta/B-cell memory and T-cell CD4 memory resting/B-cell memory ratios were up-regulated in sepsis. Similarly, it can be speculated that the decline in B-cell memory is higher than that of the other two types of cells. Furthermore, the cluster analysis revealed inherent heterogeneity in the immune cell ratios among sepsis patients, suggesting the complexity of the disease. This heterogeneity may arise from various factors, including the patient's own genetic and physiopathological characteristics, the stage of infection, and the specific type of bacterial or viral infections. Therefore, in clinical diagnosis and treatment, relying on a single immune cell or molecule can be challenging. Instead, a comprehensive consideration of multiple factors is necessary, and different treatment strategies should be formulated for patients with different characteristics. At the same time, meticulous experimental design is crucial, ensuring that factors beyond the disease are carefully controlled in the cohort analysis.

We analyzed the relationship between the differential immune cell ratios and survival and confirmed the importance of these ratios as potential cell markers or targets for subtyping and risk assessment. For example, M1 macrophages are considered to have pro-inflammatory effects in sepsis, while M1 macrophages transform into M2 is immunosuppressive and may have adverse effects on patients [33]. IL-10 has an immunosuppressive effect and can convert M1 to M2. For the first time, we found that the M1/M2 ratio exhibited prognostic significance, with a higher ratio indicating a better prognosis (Fig. 3). Activated NK cells are known to produce IFN-γ, which plays an important role in sepsis progression, such as inflammatory response and multi-organ failure [34]. However, some studies have found that NK cells have anti-infection and anti-inflammatory effects. NK activity decreases and IFN-γ production capacity is weakened in severe patients [35]. These contradictory findings may stem from small sample sizes or differences in sampling timepoints. For the first time, we found that the activated NK-cell/resting NK-cell ratio has a prognostic effect, with a higher ratio linked to a more favorable clinical outcome. Treg is considered to play an immunosuppressive role and has the effect of inhibiting the activation and proliferation of CD4 and CD8 T cells [36]. We found for the first time that the Treg/T-cell CD8 ratio can serve as a prognostic indicator, with a lower cell ratio indicating a better 28-day survival rate. Therefore, these immune cell ratios are potential cell markers or targets for subtyping or risk assessment; for example, higher expression of certain immune cell ratios has a higher risk of death.

We analyzed the relationship between the KEGG pathways and the immune cell ratios. For example, we found that neutrophil/macrophage M2 has the highest correlation with the TLR signal transduction pathway. Neutrophils, a polymorphonuclear leukocyte, are the most abundant circulating leukocyte population in the human immune system, accounting for 50–70% of all circulating leukocytes in healthy adults [37]. Patients with neutropenia are more likely to develop microbial infections. We found that the number of neutrophils was highly correlated with the TLR signal transduction pathway, and neutrophil count was positively associated with survival [38]. TLRs may contribute to the organ dysfunction and mortality that occurs in sepsis [39]. Eosinophilia occurs early in primary immunodeficiency disease [12]. For the first time, we discovered that the eosinophil/T-cell CD8 ratio was highly negatively correlated with primary immunodeficiency pathways, indicating its potential as an indicator of immunodeficiency. It has been suggested that primary immunodeficiency may be responsible for the development of fulminant sepsis [40].

At present, no research has proposed the immune cell ratios that can distinguish different types of infections. Determining the infection or not and the type of infection is the prerequisite for formulating a scientific and reasonable clinical treatment plan. We found multiple immune cell ratios that have good performance in distinguishing bacterial, viral sepsis, and SIRS, and some of them can also predict patient survival. For example, M0 macrophages were associated with a viral infection, so their decline rates may be higher than other cells (Fig. 4). In addition, resting NK cells were associated with a bacterial infection. By transcriptome analysis, we found that the macrophage marker CD14 was less up-regulated in SIRS than bacterial and viral infections and a higher macrophage M0/Tregs ratio could distinguish SIRS from other types of sepsis. Notably, traditional PCT or CRP fails to effectively distinguish these different infection types. These results suggest that clinicians should formulate differential diagnoses and treatment strategies for different sepsis subtypes or causes to improve the treatment efficacy.

Our results indicate that cell ratios may have better prognostic abilities than gene ratios or NLR. The reason may be that cell ratios use the information of multiple genes, while gene ratios only use the information of two genes. The prognostic value of NLR in sepsis has been extensively studied. However, the association between NLR and adverse outcomes of sepsis remains controversial [41]. Some studies found that the NLR is positively associated with mortality [42], some reported no associations [14, 43], and some found negative connections [44, 45]. It is thought that the discrepancies in study results are attributed to different sample sizes or other confounding parameters that may affect the association, such as measuring time at admission, the first 5 days, or the late stage of sepsis [28].

Finally, to reveal potential molecular mechanisms of the poor prognosis outcome in sepsis with high eosinophil/B.cell.memory ratio samples (Fig. 3), we performed differential analysis and gene co-expression analysis. Indeed, we found pathways that are associated with the two immune cells. For example, the interferon signaling pathway (M9) was down-regulated in high-ratio samples, which is important in B-cell activation [46]. The specific granule pathway (M26) was up-regulated in high-ratio samples, while granules are unique to eosinophils [47]. Interestingly, eosinopenia has been identified as a reliable marker of sepsis upon admission to ICUs [47]. This information indicates that our results are meaningful biologically and clinically. The immune cell ratios can be promising candidates for clinical applications.

However, it is essential to acknowledge that our findings should be further validated in future studies with large-scale clinical cohorts. Although our research provides valuable insights into the immune cell ratios and their potential implications in sepsis, the sample size in our current study is limited. Larger and more diverse clinical cohorts would enhance the robustness and generalizability of our results. This will enable us to account for potential variables and confounding factors that may exist within a more heterogeneous sample. In addition, larger cohorts would allow for subgroup analyses, enabling more in-depth exploration of specific patient characteristics or disease subtypes that could impact immune cell ratios in sepsis. Besides, the biological mechanisms of cell ratios should be investigated to confirm their roles in sepsis. Reliable cellular biomarkers should be carefully chosen for specific cell counting for real clinical applications.

In conclusion, our study is the first to characterize the expression changes of immune cell ratios in sepsis, offering cellular markers and targets for diagnostic subtyping, risk assessment, prognosis, and drug development.

### Supplementary Information

**Additional file 1. **Differential gene list and the assignment of variable genes to gene-coexpression modules for sepsis samples with high Eosinophil/B.cell.memory ratio vs. low Eosinophil/B.cell.memory ratio based on dataset GSE65682.

**Additional file 2: Figure S1.** Two cell ratios were validated in the single-cell data set. (A) Boxplot for the gene ratio CD16/CD38 in control and sepsis subjects of data set GSE185263. (B) Correlation analysis between SOFA and the gene ratio CD16/CD38 in data set GSE185263. SOFA: the sequential organ failure assessment score. (C) ROC analysis of the gene ratio CD16/CD38 in viral sepsis diagnosis in data set GSE63990. AUC: area under the curve. (D) Analysis of variance (ANOVA) of the gene ratio CD16/CD38 in different infection types, including non-infectious illness, bacterial infection, and viral infection in data set GSE63990.

## Data Availability

The data that support the findings of this study are available from the corresponding author upon reasonable request.
