# Supplementary material for: Comprehensive analysis of immune cell landscapes revealed that immune cell ratio eosinophil/B.cell.memory is predictive of survival in sepsis
Source: Eur J Med Res. 2023 Dec 5;28:565. doi: 10.1186/s40001-023-01506-8 (PMC10696691; doi:10.1186/s40001-023-01506-8)
Supplement: Supplementary file 2 — Additional file 2: Figure S1. Two cell ratios were validated in the single-cell data set. (A) Boxplot for the gene ratio CD16/CD38 in control and sepsis subjects of data set GSE185263. (B) Correlation analysis between SOFA and the gene ratio CD16/CD38 in data set GSE185263. SOFA: the sequential organ failure assessment score. (C) ROC analysis of the gene ratio CD16/CD38 in viral sepsis diagnosis in data set GSE63990. AUC: area under the curve. (D) Analysis of variance (ANOVA) of the gene ratio CD16/CD38 in different infection types, including non-infectious illness, bacterial infection, and viral infection in data set GSE63990. [file 40001_2023_1506_MOESM2_ESM.docx]

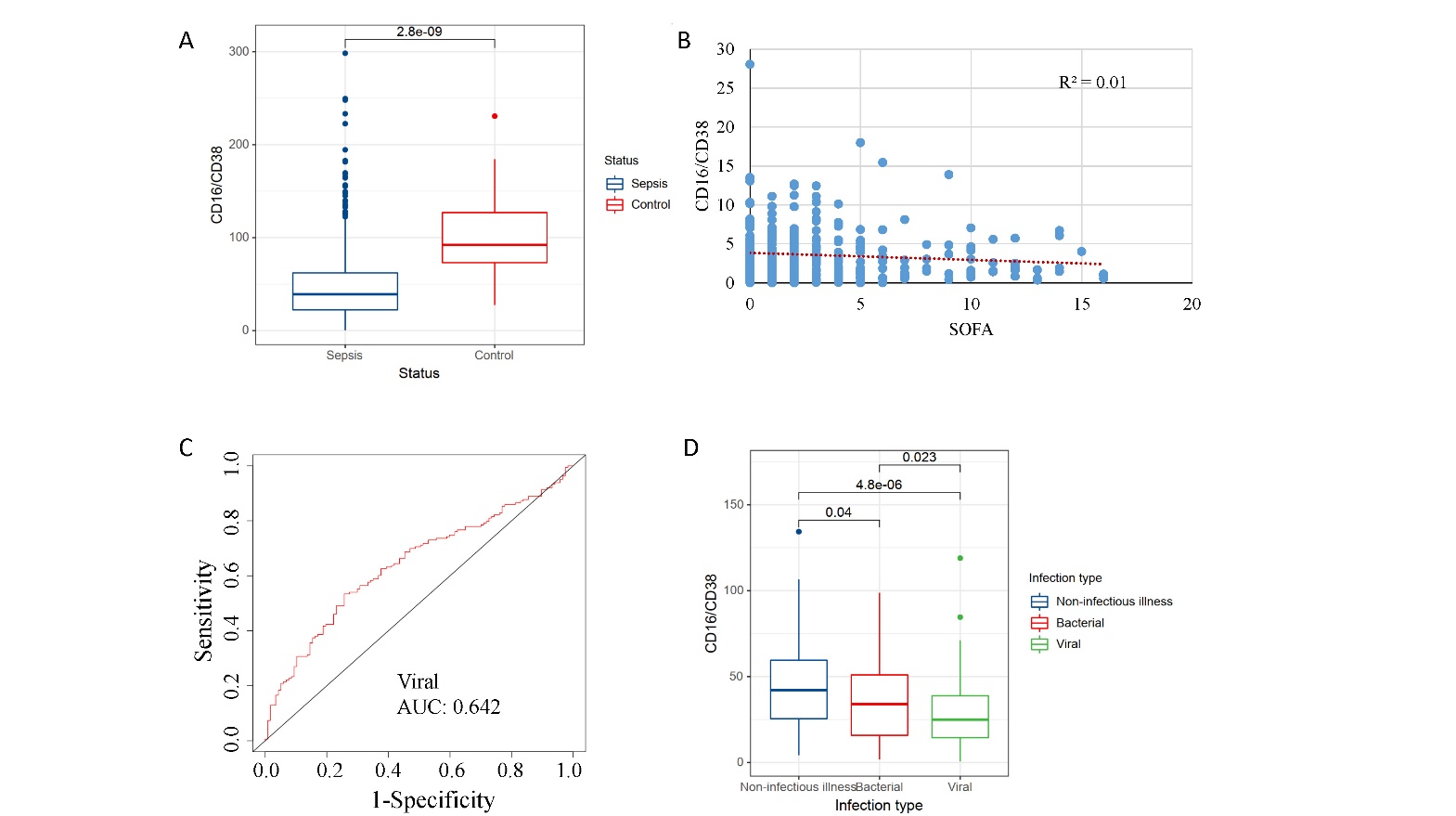


**Figure S1 Two cell ratios were validated in the single-cell dataset.** (A) Boxplot for the gene ratio CD16/CD38 in control and sepsis subjects of dataset GSE185263. (B) Correlation analysis between SOFA and the gene ratio CD16/CD38 in dataset GSE185263. SOFA: the sequential organ failure assessment score. (C) ROC analysis of the gene ratio CD16/CD38 in viral sepsis diagnosis in dataset GSE63990. AUC: area under the curve. (D) Analysis of variance (ANOVA) of the gene ratio CD16/CD38 in different infection types, including non-infectious illness, bacterial infection, and viral infection in dataset GSE63990.
